# Label-free quantitative proteomics analysis of jujube (*Ziziphus jujuba* Mill.) during different growth stages†

**DOI:** 10.1039/d1ra02989d

**Published:** 2021-06-22

**Authors:** Xiaoli Huang, Zhaohua Hou

**Affiliations:** College of Food Science and Engineering, Qilu University of Technology (Shandong Academy of Sciences) No. 3501 Daxue Road, Changqing District Ji'nan Shandong Province 250353 P. R. China kevin19820427@163.com +86 531 89631191 +86 188 66151356

## Abstract

Chinese jujube (*Zizyphus jujuba* Mill.), a member of the Rhamnaceae family with favorable nutritional and flavor quality, exhibited characteristic climacteric changes during its fruit growth stage. Therefore, fruit samples were harvested at four developmental stages on days 55 (young fruits), 76 (white-mature fruits), 96 (half-red fruits), and 116 (full-red fruits) after flowering (DAF). This study then investigated those four growth stage changes of the jujube proteome using label-free quantification proteomics. The results identified 4762 proteins in the samples, of which 3757 proteins were quantified. Compared with former stages, the stages examined were designated as “76 *vs.* 55 DAF” group, “96 *vs.* 76 DAF” group, and “116 *vs.* 96 DAF” group. Gene Ontology (GO) and KEGG annotation and enrichment analysis of the differentially expressed proteins (DEPs) showed that 76 *vs.* 55 DAF group pathways represented amino sugar, nucleotide sugar, ascorbate, and aldarate metabolic pathways. These pathways were associated with cell division and resistance. In the study, the jujube fruit puffing slowed down and attained a stable growth stage in the 76 *vs.* 55 DAF group. However, fatty acid biosynthesis and phenylalanine metabolism was mainly enriched in the 96 *vs.* 76 DAF group. Fatty acids are precursors of aromatic substances and fat-soluble pigments in fruit. The upregulation of differential proteins at this stage indicates that aromatic compounds were synthesized in large quantities at this stage and that fruit would enter the ripening stage. During the ripening stage, 55 DEPs were identified to be involved in photosynthesis and flavonoid biosynthesis in the 116 *vs.* 96 DAF group. Also, the fruit entered the mature stage, which showed that flavonoids were produced in large quantities. Furthermore, the color of jujube turned red, and photosynthesis was significantly reduced. Hence, a link was established between protein profiles and growth phenotypes, which will help improve our understanding of jujube fruit growth at the proteomic level.

## Abstract 2

Chinese jujube (*Zizyphus jujuba* Mill.), a member of the Rhamnaceae family with favorable nutritional and flavor quality, exhibited characteristic climacteric changes during its fruit growth stage.

## Graphical abstract

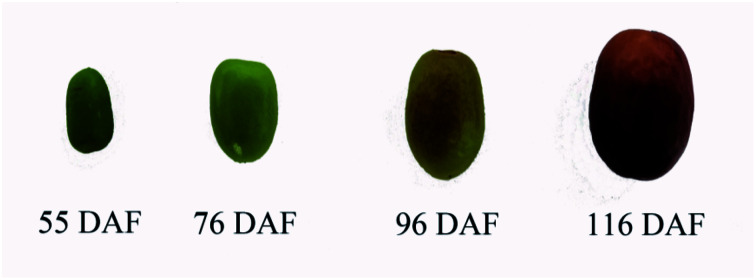

## 1 Introduction

Chinese jujube (*Zizyphus jujuba* Mill.), a highly nutritious fruit, is one of the most popular fruits in China. About 6.25 million tons of jujube dry fruit are produced annually from a cultivated area of approximately 2 million ha.^1^ Recent studies have revealed that its fruits contain various functional compounds such as vitamin C, amino acids, triterpene acids, polysaccharides, and polyphenols.^2–4^ Based on bioactivity analyses, jujube fruit has been shown to lower blood pressure, reverse liver disease, treat anemia, and inhibit the growth of tumor cells.^5^ Additionally, several reports have demonstrated the neuroprotective activities^6^ and blood-nourishing function of jujube fruit.^7^

Jujube exhibits characteristic climacteric changes during its fruit growth stages. During this stage, the jujube fruit cells divide quickly, and the number of cells increases rapidly; however, the cell volume increases slowly. Then, the volume of jujube fruit cells rapidly expands and continues to mature until the jujube reaches the white period. During the ripening process, owing to changes in the levels of flavonoids, carotenoids, and chlorophyll,^8^ jujube fruits pericarp color changes from green to white during early ripening and later turns red. Simultaneously, aromatics or characteristic components are produced in large numbers. During fruit development and ripening, the epigenetic characteristics of jujube fruit, such as color changes, sugar metabolism, cell wall restructuring, stress response, and aroma volatile synthesis, are involved in a series of physiological metabolic processes.^9^

Recently, studies on jujube fruit focused on biochemical changes, including its metabolic and transcriptional regulation. Observing the physiological changes occurring in this fruit is a common method for studying the process of fruit growth and change. Li *et al.* reported how the skin color of jujube fruit during maturation was due to changes in the levels of flavonoids, carotenoids, and anthocyanins.^8^ Metabolomic profiling also reflected the genetic, epigenetic, and environmental factors that influenced its cellular physiology. Similarly, Shi *et al.* reported that the *ZjANS* and *ZjUGT*79*B*1 promoters were activated by *ZjMYB*5, *ZjTT*8, and *ZjWDR*3 proteins, which regulated anthocyanin biosynthesis in jujube fruit skins.^10^ Transcriptomic profiling is another promising approach to analyze the entire genome, which provides details regarding the biological processes underlying jujube fruit development. Liu *et al.* conducted transcriptome analysis to investigate the changes in gene expression in surface-pitted jujube fruit.^11^ Furthermore, Chen *et al.* found that 39 *ZjWRKY* genes were expressed during jujube fruit development and ripening.^12^ These studies have provided an enormous amount of data that expand our knowledge of the molecular events associated with ripening. Proteomic profiling bridges data-rich information regarding alterations of the proteome that occur because of the transcriptome, and metabolome has been used as well to analyze expressed proteins and protein function in a cellular context. Research on fruit proteomics, such as that for peaches,^13^ tomatoes,^14^ apples,^15,16^ and muskmelons,^17^ has also shown that proteomic analysis approaches can illustrate proteins involved in fruit ripening and senescence.

Proteomics is therefore a powerful method for exploring differential proteins and their complex regulatory mechanisms.^18^ Furthermore, the label-free quantitative (LFQ) proteomics approach is a mass-based quantitative technique for identifying and quantifying proteins.^19^ Compared with proteome quantitation based on labeling techniques, LFQ can detect a large number of samples at a lower cost and ease. LFQ also has a simpler experimental workflow.^20,21^ However, quantitative analysis is comparatively time-consuming, as no multiplexed analysis is possible as in the case of label-free proteomics. Additionally, the instrumentation time necessary for LFQ analysis is significantly longer than that of the label-based quantitative method, and variations during sample preparation, including chromatography and MS acquisition, are increased.^22^ Furthermore, the saturation of signals at high protein abundance levels can be due to limitations in the ion-trapping capacity, duty cycle, or ionization efficiency of the mass spectrometer.^23^ Therefore, Venable *et al.* suggested data-independent analysis (DIA) as an alternative to data-dependent analysis (DDA).^24^ DIA increases the signal-to-noise effects during analysis by threefold to fivefold and identifies undetected peptides in the parent ion scan of a typical DDA experiment.^25^ LFQ approaches are also suitable for comparing protein expression levels between different conditions and provide good approximations of absolute protein abundance, especially when used with carefully designed experiments and relevant statistical methods.^21^ Interestingly, LFQ has recently been used in numerous plant proteomics studies, such as in apricot,^19^ strawberry,^26^ tomato,^14^ apple,^27^ and citrus.^28,29^

In this research, fresh jujube were collected at four stages of development and were extracted to analyze its crude proteins using nanoflow liquid chromatography-tandem mass spectrometry (nano LC-MS/MS) instruments. The four growth stage changes of jujube proteome were also studied using LFQ to investigate the differentially expressed proteins (DEPs) involved in significant metabolic changes during development and ripening. Next, we used bioinformatics to investigate ripening-related pathways involved in significant proteome changes during jujube ripening.

## 2 Material and method

### 2.1 Material and treatments

**Fig. 1:**
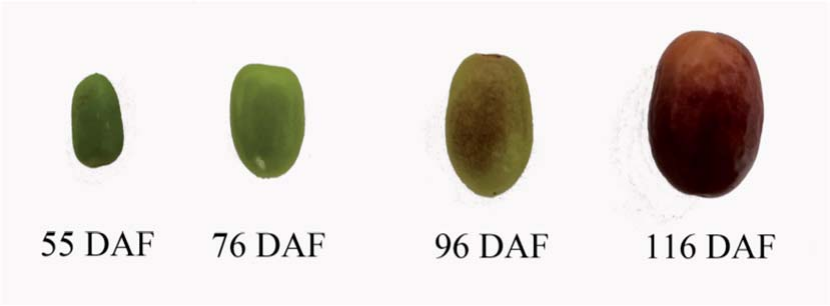
Jujube in different developmental and ripening stages.

Fresh jujube, “Xinxing,” was obtained from a local farm in Binzhou, Shandong, China, in 2020. Young fruits (55 days after flowering [DAF]), white-mature fruits (76 DAF), half-red fruits (96 DAF), and full-red fruits (116 DAF) of Xinxing were collected separately from jujube trees (Fig. 1).^8,30^ Pest- and disease-free jujube fruits with uniform size, having no mechanical damage, were used for the experiments. The jujube pulp was then frozen in liquid nitrogen and pulverized. Next, the powdered samples were stored in a refrigerator at −80 °C. About 70–80 fruits were picked during each stage randomly and then divided into three biological replicates.

### 2.2 Proteomic analysis

#### 2.2.1 Protein extraction

The method of protein extraction referenced previous studies and slightly altered.^19,31^ Approximately 3 g of the powdered sample was thoroughly homogenized in 15 mL pre-cooling acetone containing a trichloroacetic acid mixed solvent (trichloroacetic acid/acetone, 1 : 9 [v/v], containing 0.07% β-mercaptoethanol) at 5000 rpm for 60 s. Then, the mixture was placed at −20 °C for 4 h, and centrifuged at 12 000 × *g* for 15 min at 4 °C, after which the supernatant was discarded. Next, pellets were resuspended in pre-cooling acetone and washed three times, after which the precipitation was air-dried. Subsequently, crude proteins were divided by adding 15 volumes of urea extraction buffer (8 M urea, 0.1 M Tris–HCl, pH 8.5) and sonicated for 5 min (pulse 2 s on, 3 s off, 50% amplitude) in an ice-water bath (Sonics® Vibra-Cell VCX 130). After, centrifugation was conducted at 12 000 × *g* for 15 min, and the supernatant was filtered with 0.45 μm filters. Bovine serum albumin was used as a reference substance, and Coomassie Brilliant Blue G-250 as the chromogenic agent. Also, visible spectrophotometry was used to determine the content of protein in the filtrate by UV at 595 nm.

#### 2.2.2 Filter-aided sample preparation digestion

Protein extracts were digested with trypsin following the filter-aided sample preparation procedure.^31,32^ Briefly, total-protein samples (200 μg) were loaded into filtration devices (10 kD Microcon, Millipore) and centrifuged at 14 000×*g* for 15 min. Then, detergent, DTT, and other low-molecular-weight components were removed using urea extraction buffer by repeated ultrafiltration. After centrifugation, concentrates were reduced with 100 μL of 100 mM DTT, containing 50 mM NH_4_HCO_3_ (50 °C, 30 min). Next, 100 μL of 100 mM iodoacetamide in a urea extraction buffer was added to the block-reduced cysteine residues. The samples were incubated for 40 min in darkness. Subsequently, the filters were washed with 100 μL of urea extraction buffer three times and then with 100 μL of 25 mM NH_4_HCO_3_ buffer twice. Finally, the concentrate was digested at 37 °C for 16 h with 200 μL trypsin (Promega), after which the resulting peptides were collected as filtrates. Eluted peptides were subsequently acidified with TFA to a final concentration of 0.1% and cleaned up using PepClean™ C-18 Spin Columns (Pierce) according to the manufacturer's instructions.

#### 2.2.3 Mass spectrometry analysis and protein identification

The method of mass spectrometry analysis and protein identification referenced previous studies and slightly altered.^33^ The digested peptides were analyzed on a Q-Extractive HF mass spectrometer (Thermo Scientific, Waltham, MA, USA) coupled with an online nano LC-MS/MS on an Ultimate 3000 system (Dionex). LC was then used to separate collected fractions. Briefly, the peptide mixture was loaded on a reversed-phase trap column (Thermo Scientific Acclaim PepMap 100, 100 μm × 2 cm, nanoViper C_18_) connected to a C-18 reversed-phase analytical column (Thermo Scientific Acclaim PepMap 100, 15 cm long, 75 μm inner diameter, 3 μm resin). Then, buffer A (0.1% formic acid) was used in the separation, with a linear gradient of buffer B (80% acetonitrile and 0.1% formic acid) at a flow rate of 300 nL min^−1^. The linear gradient was processed as follows: 4–40% solution B for 98 min, 40–99% solution B for 5 min, and 99% solution B for 5 min. Furthermore, MS data were acquired using a data-dependent top 20 method, by dynamically choosing the most abundant precursor ions from the survey scan (*m*/*z* 150–2000) for higher-energy collisional dissociation (HCD) fragmentation. The instrument parameters were set as follows: automatic gain control target, 3e;^6^ dynamic exclusion duration, 30 s; resolution for survey scans, 120 000 at *m*/*z* 200; resolution for HCD spectra, 30 000 at *m*/*z* 200; and isolation width, *m*/*z* 2. The normalized collision energy was 27, and the underfill ratio was 0.1%. Dynamic exclusion was employed again for 30 s to prevent repetitive selection of peptides. Each sample was analyzed in three technical triplicates.

#### 2.2.4 Protein identification

Raw data from mass spectrometric analysis were analyzed using Proteome Discoverer software (ver. 2.2, Thermo Scientific, Bremen, Germany). Search parameters were precursor error tolerance (10 ppm) and fragment ion tolerance (0.5 Da). The peptide section was greater than six amino acids in length. Tryptic cleavage was then selected, and up to two missed cleavages were allowed. Carbamido methylation on cysteine was also set as a fixed modification, and oxidation on methionine was assigned as a variable modification parameter. For protein identification, searches were conducted against the UniProtKB/Swiss-Prot *ziziphus jujuba* database (release 2020_12, 35 660 total entries, downloaded 12/26/20) using the Sequest HT search engine. The false discovery rate of less than 0.01 and relaxed criteria of 0.05 was used. Razor and unique peptides were also used for protein quantification.

#### 2.2.5 Data analysis and statistical analysis

Data were analyzed using Perseus (ver.1.6.5.0) software and the “Wu Kong” platform powered by R language (https://www.omicsolution.com/wkomics/main/).^34^ Groups with values greater than or equal to 2/3 were included. Missing values left were imputed using the KNN method. The *t*-test was then performed to analyze the differences between two groups, whereas a one-way analysis of variance test was used among more than three groups. To analyze significant differences between the quantitative results, at least two non-null data from triplicated experimental data in the same sample group were statistically analyzed. log2 intensity values that were missing in one sample out of the groups were also inputted using a downshifted normal distribution with width 0.3 and downshift 1.8 for each sample. During the screening of DEPs, we used *p*-value < 0.01 and |log2 (fold change)| > 1 as the threshold criteria to determine the significance of differences between protein expression levels.

Principal component analysis (PCA) is a common method for analyzing data dimension reduction. This method can reduce the dimension of the data and keep the feature that contributes most to the variance. It is equivalent to keeping the low-order principal components and ignoring the higher-order ones. Hierarchical cluster analysis (HCA) was set as the distance method for rows and columns was Euclidean, and the clustering method was complete.

### 2.3 Bioinformatic analysis

The altered abundance of proteins was further analyzed using bioinformatic methods such as Gene Ontology (GO) functional annotation, KEGG pathways, and protein–protein interaction (PPI) network predictions as described previously.^32,35^ Briefly, GO annotation, KEGG pathway, and PPI analyses were carried out using the online software OmicsBean (www.omicsbean.cn).^36^ The generated data were mapped to *Arabidopsis thaliana*. PPI information of the studied proteins were also retrieved from the IntAct molecular interaction database (http://www.ebi.ac.uk/intact/) using their gene symbols. The PPI prediction network was also analyzed using the online software STRING version 11.0 (http://string-db.org/) and revised using Cytoscape version 3.7.2.^37^ Additionally, the Molecular Complex Detection (MCODE) plugin in Cytoscape version 3.7.2, was used to screen modules of the PPI network (degree cutoff = 2, node score cutoff = 0.2, *k*-core = 2, and max. depth = 100).

## 3 Results

### 3.1 Data analysis

#### 3.1.1 Protein identification and quantification

**Fig. 2:**
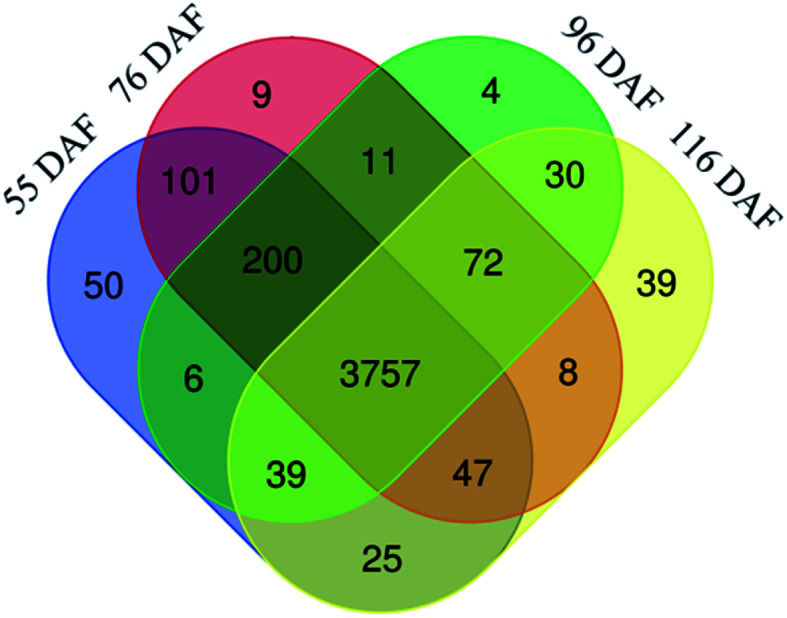
Venn diagram of the number of different proteins identified in the jujubes fruit proteome in four growth stages.

As shown in Fig. 2, 4762 master proteins were identified, and 3757 were quantified. Also, 4225, 4205, 4119, and 4017 proteins were identified in 55, 76, 96, and 116 DAF, respectively. Additionally, 3757 proteins were identified in all growth stages, accounting for about 79% of all identified proteins (Fig. 2). Furthermore, the results showed that the physiological and biochemical processes were similar in the four developmental and ripening stages of jujube.

#### 3.1.2 PCA and hierarchical cluster analysis of proteins

**Fig. 3:**
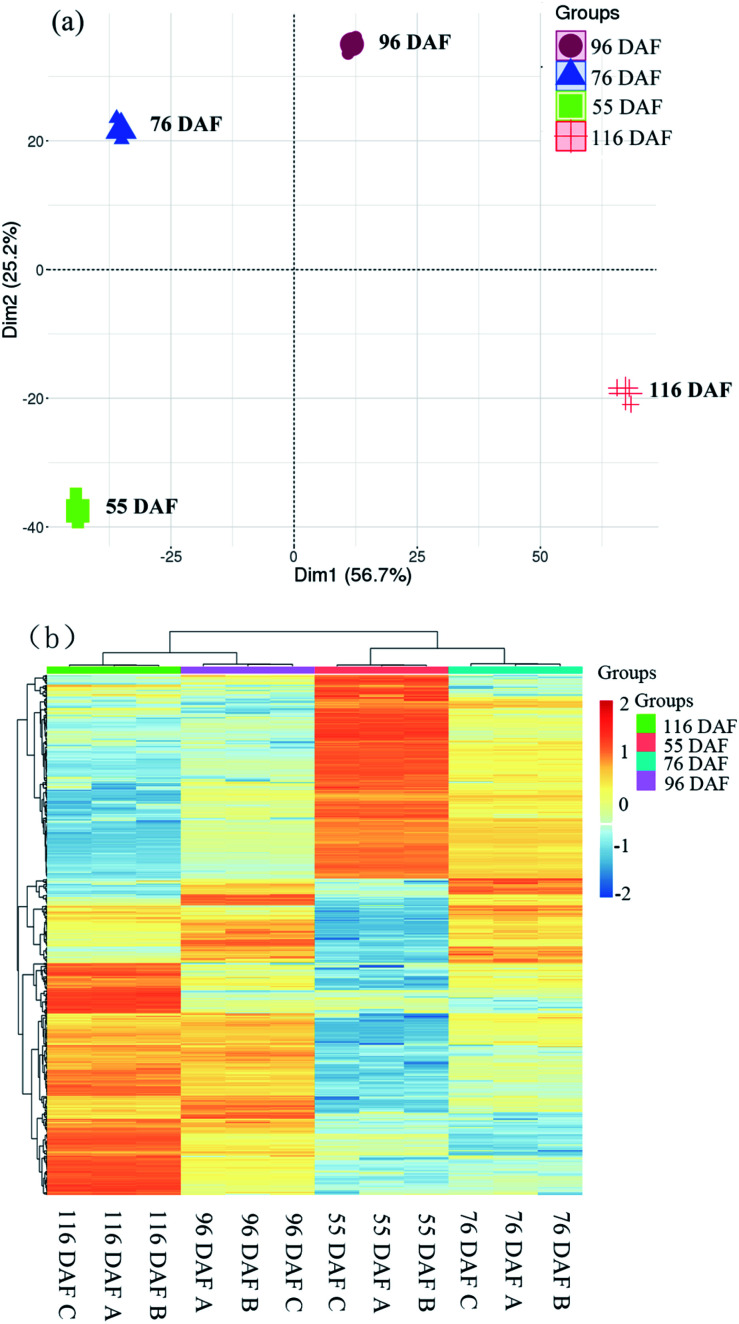
Dynamic proteome of jujube development and ripening: (a) principal component analysis of proteomics data from four developmental stages of jujube fruit. (b) Clustering analysis was performed to display the significant differentially expressed proteins during the development of jujube fruit. Red represents high expression, while green represents low expression.

As a dimensionality reduction method, unsupervised PCA effectively represented the original data using fewer dimensions, retained the distribution characteristics, and maximized the variance of data in each dimension.^38^ The results of the PCA and HCA of proteins are shown in Fig. 3.

The score plot of PCA showed that the first two principal components explained 81.9% of the variability in the data, which each accounted for 56.7% and 25.2% of the total variance. Cluster analysis of the different samples also revealed that 116 and 96 DAF were aggregated into a cluster, and 76 and 55 DAF into another cluster. Overall, the results of the statistical analysis of the heat map and PCA were highly consistent, indicating that the objects to be analyzed can be divided into two clusters. Results showed that the individuals within the clusters had high similarity and that the individuals between clusters had great differences. It was therefore concluded that the samples can be divided to distinguish among these jujube samples. Therefore, samples belonging to 116 and 96 DAF were placed at the ripening stage, and those under 76 and 55 DAF were placed at the growth stage.

#### 3.1.3 Differentially expressed proteins

**Fig. 4:**
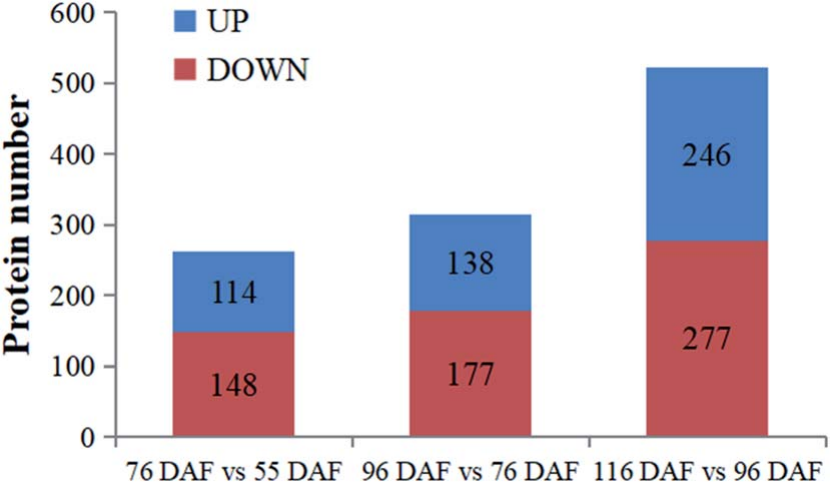
Identified proteins and differentially expressed proteins at four developmental and ripening stages of jujube fruit.

DEPs at different stages (55, 76, 96, and 116 DAF) of jujube fruits were compared and analyzed. Three groups were obtained: “76 *vs.* 55 DAF,” “96 *vs.* 76 DAF,” and “116 *vs.* 96 DAF”. DEPs are illustrated in Fig. 4.

### 3.2 Bioinformatics analysis

#### 3.2.1 Functional annotation and enrichment analysis of GO

For insight into the functional categories that were altered in the different stages, three groups of DEPs were annotated and enriched in the GO database, to the biological process (BP), cell components (CC), and molecular function (MF).

**Fig. 5:**
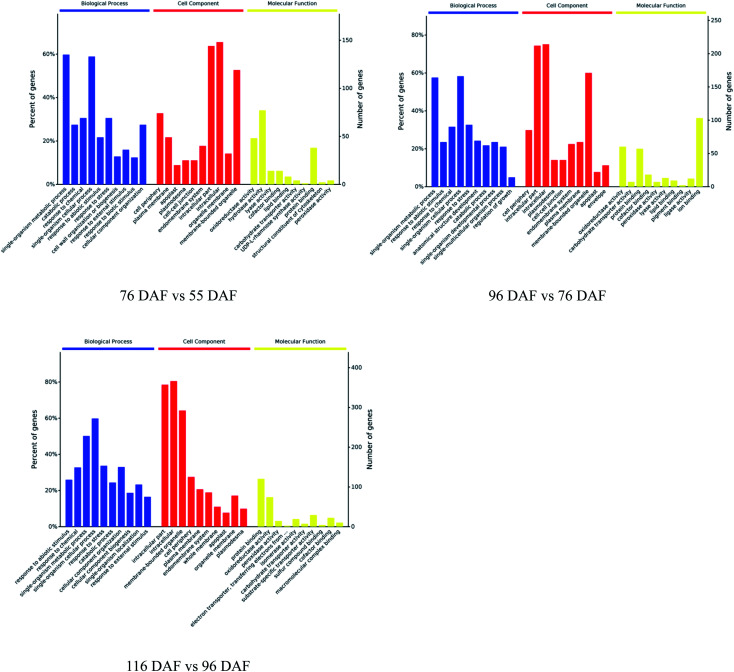
Gene ontology functional classification analysis of proteins with different developmental and ripening stages of jujubes (*p*-value < 0.01).

The proteins identified and sorted by *p*-value were then classified into three types, namely, CC, MF, and BP (Fig. 5 and ESI Table 1†). From the results, proteins of the 76 *vs.* 55 DAF group in the BP category were mainly related to single-organism metabolic processes (56%), catabolic processes (6%), response to chemicals (8%), single-organism cellular processes (5%), and cell wall organization or biogenesis groups (2%). Cellular components that were over-represented among proteins with increased abundance were the cell periphery (31%), apoplast (3%), and plasmodesma (2%). Furthermore, oxidoreductase (20%), hydrolase (32%), lyase (3%), and lipid-binding (2%) activities were significantly enriched in the GO-MF class.

In the 96 *vs.* 76 DAF group, the proteins mainly identified in the BP category were those related to single-organism metabolic processes (56%), response to abiotic stimulus (9%), response to chemicals (4%), and single-organism cellular processes (5%). Similarly, cellular components that were over-represented among proteins with increased abundance were the cell periphery (29%) and intracellular parts (52%). Furthermore, MFs that were significantly enriched were related to oxidoreductase (21%), carbohydrate transporter (2%), and protein-binding (16%) activities.

In the 116 *vs.* 96 DAF group, proteins mainly identified in the BP category were related to responses to abiotic stimulus (24%), response to chemicals (14%), single-organism metabolic processes (22%), and single-organism cellular processes (9%). Similarly, cellular components that were significantly enriched were related to intracellular parts (73%) and the cell periphery (7%). Meanwhile, two sub-categories were mainly related to the GO-MF class, such as protein-binding (24%) and oxidoreductase (19%) activities.

The three most enriched GO terms for DEPs in jujube are listed in Table 1. GO annotation showed that cell-wall-related categories, such as the “plasma membrane,” “apoplast,” and “oxidoreductase activity,” were significantly enriched GO terms in the 76 *vs.* 55 DAF group. It was also suggested that the formation of cell walls played a vital role in this stage. Moreover, in the 96 *vs.* 76 DAF group, the “intracellular” and “protein binding” enriched terms from the CC and MF ontology, respectively, inferred that identified proteins were those found mainly in the cell and referred to the ability of proteins to bind to other substances. Additionally, the “response to the abiotic stimulus” pathway was enriched in BP, which shows that jujube proteins were stimulated by a large number of external environmental factors from 76 to 96 days. In the 116 *vs.* 96 DAF group, the “intracellular organelle part,” “protein-binding” and “response to abiotic stimulus” pathways were enriched. This result proposes that most of the metabolic activity in this group was concentrated in the organelle and that proteins such as carrier proteins were involved in the transport of substances.

**Table 1:** KEGG pathway of differential expression proteins in 76 *vs.* 55 DAF

Map name (Map ID)	Gene name	Uniprot ID	Protein name/description	Fold change
**76 DAF *vs.* 55 DAF**				
Amino sugar and nucleotide sugar metabolism	*ASD*1	A0A6P4A4X6	Non-reducing end alpha-l-arabinofuranosidase	3.33
	*At*5*g*28840	A0A6P3ZJY5	GDP-mannose 3,5-epimerase 2	0.16
	*RGP*3	A0A6P4AEG0	UDP-arabinopyranose mutase	0.46
	*APS*1	A0A6P4ATW6	Glucose-1-phosphate adenylyltransferase	0.41
	*UXS*2	A0A6P4AAY8	UDP-glucuronic acid decarboxylase 2-like	0.33
	*NRS*/*ER*	A0A6P3YZ71	Bifunctional dTDP-4-dehydrorhamnose 3,5-epimerase/dTDP-4-dehydrorhamnose reductase	0.39
	*BXL*5	A0A6P4ADV0	Probable beta-d-xylosidase 5	0.34
	*HEXO*3	A0A6P3Z8D1	Beta-hexosaminidase	0.50
	*At*1*g*47840	A0A6P4BUB5	Phosphotransferase	0.23
	*RHM*1	A0A6P3YZ77	Trifunctional UDP-glucose 4,6-dehydratase/UDP-4-keto-6-deoxy-d-glucose 3,5-epimerase/UDP-4-keto-l-rhamnose-reductase RHM1-like	0.35
	*MUR*4	A0A6P4BDD1	UDP-arabinose 4-epimerase 1 isoform X1	0.23
Ascorbate and aldarate metabolism	At5g28840	A0A6P3ZJY5	GDP-mannose 3,5-epimerase 2	0.16
	ALDH3H1	A0A6P3ZVT6	Aldehyde dehydrogenase	5.00
	ALDH2B7	A0A6P4ADF3	Aldehyde dehydrogenase family 2 member B7, mitochondrial isoform X1	2.02
	APX3	A0A6P4A7X1	l-Ascorbate peroxidase	0.48
	At5g21105	A0A6P3Z8E5	l-Ascorbate oxidase	0.32
	MDAR1	A0A6P4AGJ0	Monodehydroascorbate reductase-like	2.31

**96 DAF *vs.* 76 DAF**				
Fatty acid biosynthesis	*FAB*2	A0A6P3Z4Y9	Acyl-[acyl-carrier-protein] desaturase, chloroplastic-like	6.51
	*CAC*2	A0A6P4A1C9	Biotin carboxylase	2.61
	*LACS*2	A0A6P6FQQ8	Long chain acyl-CoA synthetase 2 isoform X2	25.43
	*S-ACP-DES*5	A0A6P3Z904	Acyl-[acyl-carrier-protein] desaturase	2.12
	*BCCP*2	A0A6P3ZJ05	Biotin carboxyl carrier protein of acetyl-CoA carboxylase	2.95
	*CAC*3	A0A6P6GM35	Acetyl-CoA carboxytransferase	2.09
	*KAS*2	A0A6P6GDA6	3-Oxoacyl-[acyl-carrier-protein] synthase II, chloroplastic-like isoform X1	10.39
	*At*1*g*24360	A0A6P4AI21	4-Oxoacyl-[acyl-carrier-protein] reductase	2.75
	*FATA*	A0A6P4B276	Acyl-[acyl-carrier-protein] hydrolase	4.07
	*EMB*3147	A0A6P3ZAJ2	[Acyl-carrier-protein] *S*-malonyltransferase	2.64
	*ACC*1	A0A6P3ZVF1	Acetyl-CoA carboxylase	0.46
Phenylalanine metabolism	*ASP*5	A0A6P3ZTV0	Aspartate aminotransferase	3.16
	*At*4*g*12290	A0A6P4A027	Amine oxidase	2.05
	*PAL*1	A0A6P3YRX7	Phenylalanine ammonia-lyase	0.29
	*CCOAOMT*1	A0A6P3YZL7	Caffeoyl-CoA *O*-methyltransferase	2.40
	*At*3*g*15290	A0A6P4A1R3	Uncharacterized protein LOC107405566	3.01

**116 DAF *vs.* 96 DAF**				
Photosynthesis	*DRT*112	A0A6P4AGG8	Plastocyanin	0.45
	*PSBS*	A0A6P3ZYH8	Photosystem II 22 kDa protein, chloroplastic	0.40
	*PSBR*	A0A6P4A3U1	Photosystem II 10 kDa polypeptide, chloroplastic	0.37
	*petC*	A0A6P4A5F5	Plastoquinol-plastocyanin reductase	0.26
	*psaC*	A0A192AD84	Photosystem I iron-sulfur center	0.46
Flavonoid biosynthesis	*CHI*1	A0A6P3ZDQ5	Chalcone-flavonone isomerase family protein	0.11
	*DFRA*	A0A6P3ZX82	Dihydroflavonol 4-reductase-like	0.13
	*CHS*	A0A6P4A361	Chalcone synthase 1-like	0.49
	*BAN*	A0A6P4AK17	Anthocyanidin reductase ((2*S*)-flavan-3-ol-forming)	0.30
	*CYP*73*A*5	A0A6P3Z1V2	*trans*-Cinnamate 4-monooxygenase-liketrans-cinnamate 4-monooxygenase-like	0.14

#### 3.2.2 Distribution of enriched KEGG pathway

**Fig. 6:**
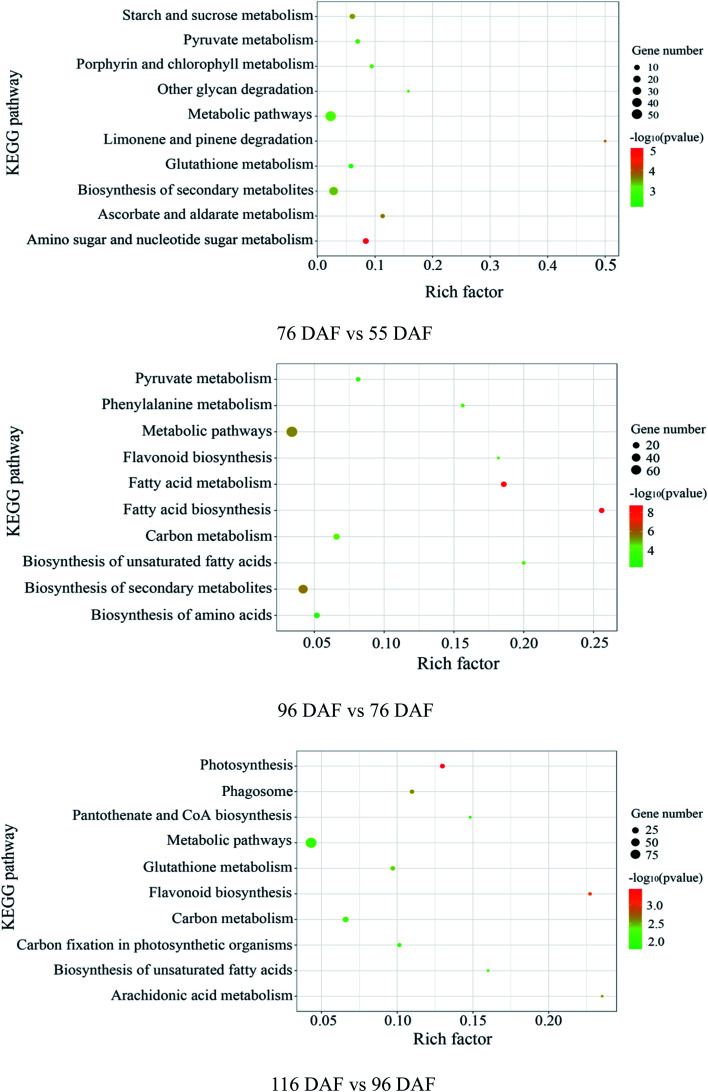
Pathway analysis, according to the Kyoto Encyclopedia of Genes and Genomes (KEGG) database: “Rich factor” means the ratio of the number of differentially expressed genes under this pathway term to the number of annotated genes under the pathway term. The larger the value is, the greater the enrichment degree is.

KEGG enrichment showed that many pathways were involved in the developmental and ripening stages of jujube fruit. Results from the DEPs in the 76 *vs.* 55 DAF group were mainly enriched in “amino and nucleotide sugar metabolism,” including “ascorbate and aldarate metabolism” (Fig. 6A), which shows that primary metabolic proteins in this group were involved in cell wall formation, consistent with the GO enrichment analysis results. Furthermore, 11 DEPs were identified in the amino and nucleotide sugar metabolism, 10 were downregulated, and only 1 was upregulated (Table 1). However, most were significantly enriched in fatty acid biosynthesis and phenylalanine metabolism in the 96 *vs.* 76 DAF group (Fig. 6B). Additionally, 11 DEPs were identified to be related to fatty acid biosynthesis, of which 10 were upregulated and only 1 was downregulated. In phenylalanine metabolism, five DEPs were identified as well, of which one was downregulated and others were upregulated (Table 1). Two biosynthetic pathways, called photosynthesis and flavonoid biosynthesis-related pathways (Fig. 6C), were enriched in the 116 *vs.* 96 DAF group, which indicated that they were involved with changes in fruit color. Similarly, five DEPs were also identified in photosynthesis, all of which were downregulated. The same is true for flavonoid biosynthesis (Table 1).

#### 3.2.3 Protein–protein interaction

**Fig. 7:**
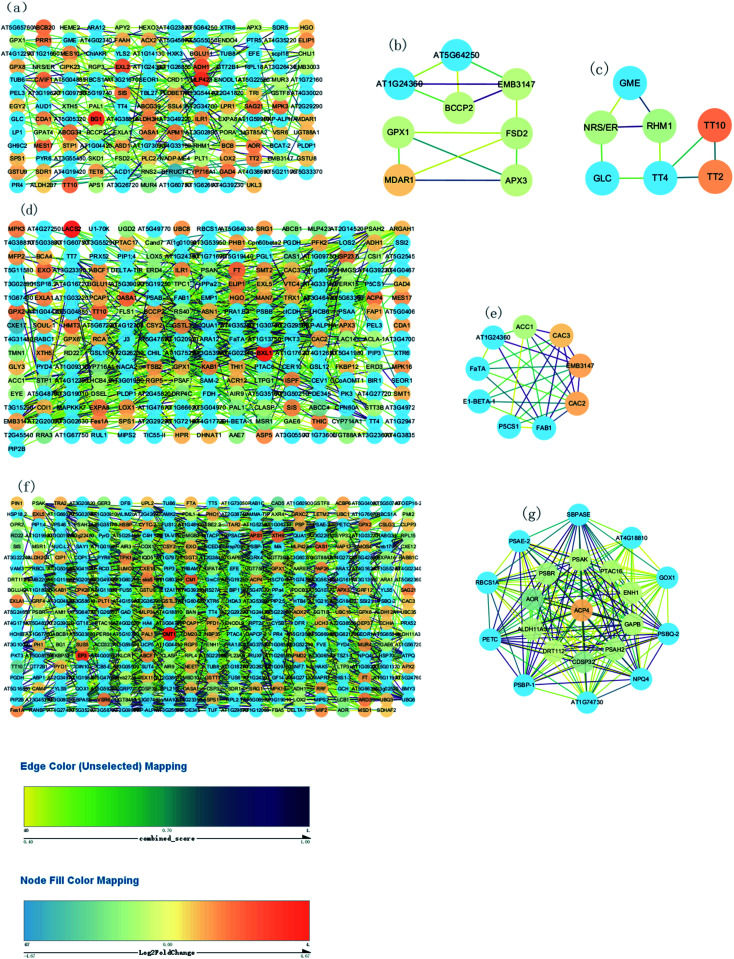
Construction of the PPI network. (a) Construction of the PPI network of “76 *vs.* 55 DAF” (b and c) MCODE analysis of the entire PPI network identified two modules in “76 *vs.* 55 DAF” (module 3 score = 3.714, module 4 score = 3). (d) Construction of the PPI network of “96 *vs.* 76 DAF.” (e) MCODE analysis of the entire PPI network identified two modules in “96 *vs.* 76 DAF” (module 2 score = 7). (f) Construction of the PPI network of “116 *vs.* 96 DAF.” (g) Construction of the PPI network of “96 *vs.* 76 DAF.” (g) MCODE analysis of the entire PPI network identified two modules in “116 *vs.* 96 DAF” (module 1 score = 19.9).

The functional significance of a protein can be predicted on the basis of its neighboring interacting partners.^39^ PPIs have led to the understanding of protein complexes and cellular protein functions through transient or stable interactions.^40^ Therefore, to predict assumed functional relationships between these DEPs, a PPI of all significantly different proteins identified at three groups was constructed and evaluated (Fig. 7).

Fig. 7A, D and F show respectively the PPI in the 76 *vs.* 55 DAF, 97 *vs.* 76 DAF, and 116 *vs.* 96 DAF groups. While the network contains 154 edges and 239 nodes in the 76 *vs.* 55 DAF group, it contains 241 edges and 651 nodes in the 96 *vs.* 76 DAF group. However, the network contains 379 edges and 1457 nodes in the 116 *vs.* 96 DAF group.

The entire PPI network was further analyzed using MCODE. The hub molecules assisted in improving the understanding of key molecular mechanisms underlying the growth of jujube fruit. From the results, 1 of 11 modules were identified (module 3 score = 3.714, module 4 score = 3; Fig. 7B–D) in the 76 *vs.* 55 DAF group. Hub module 3 also included eight hub genes: *GPX*1, *BCCP*2, *At*1*g*24360, *FSD*2, *EMB*3147, *MDAR*1, *At*5*g*64250, and *APX*3. However, hub module 4 comprised seven hub genes: *RHM*1, *GLC*, *TT*2, *GME*, *NRS*/*ER*, *TT*10, and *TT*4. Furthermore, one of the four modules were found (module 2 score = 7; Fig. 7E) in the 96 *vs.* 76 DAF group. Hub module 2 also comprised nine hub genes: *P*5*CS*1, *FaTA*, *At*1*g*24360, *EMB*3147, *CAC*3, *CAC*2, *ACC*1, *FAB*1, and *E*1-*BETA*-1. Similarly, one of the six modules were identified as well (module 1 score = 19.9; Fig. 7G) in the 116 *vs.* 96 DAF group. Hub module 1 comprised 21 hub genes: *At*4*g*18810, *ACP*4, *PSBQ*-2, *PSAE*-2, *PETC*, *PSBR*, *CDSP*32, *At*1*g*74730, *SBPASE*, *RBCS*1*A*, *PSAH*2, *NPQ*4, *PTAC*16, *GAPB*, *PSAK*, *GOX*1, *AOR*, *DRT*112, *ENH*1, *ALDH*11*A*3, and *PSBP*-1.

## 4 Discussion

Jujube fruit undergoes a rapid developmental and ripening process that involves several differential expression proteins. In this study, proteins identified were related to fruit enlargement, aroma formation, and color change. These DEPs were also enriched in many pathways such as amino and nucleotide sugar metabolism, including ascorbate and aldarate metabolism, fatty acid biosynthesis, phenylalanine metabolism, photosynthesis, and flavonoid biosynthesis.

### 4.1 Proteins associated with cell wall metabolism

In early developmental stages, fruit growth results from cell division, after which the fruit produced continues growing through cell expansion. The enlargement of the jujube fruit is normally completed before its color transition. During plant growth, the increase in cell number and volume is accompanied by changes in xyloglucan content and cell wall remodeling. However, the plant cell wall is a complex network structure containing cellulose, hemicellulose, pectin, and a small number of structural proteins.^41^ Therefore, many kinds of monosaccharides are present in cell wall polysaccharides, such as arabinose (Ara), rhamnose (Rha), mannose (Man), and xylose (Xyl), which can be linked in multiple positions to make them multipurpose cell wall building materials. Of these sugar units, ascorbate is among the most effective reducing agents in organisms, playing key roles in mitosis and cell growth.^42,43^ Ascorbate also acts as a reducing and chelating agent and scavenges free radicals. It is an important ingredient of the antioxidative defense mechanism in cells and tissues as well.^44^ Research shows that antioxidant activity gradually reduces throughout fruit development.^8^

The amino and nucleotide sugar metabolic pathways (ESI Fig. 1†), including ascorbate and aldarate metabolic pathways (ESI Fig. 2†), were associated with fruit enlargement and were enriched in the 76 *vs.* 55 DAF group. Results from the KEGG annotation and enrichment analysis showed increased expression of alpha-l-arabinofuranosidase in 76 DAF. Furthermore, it is the lowly expression of a UDP-arabinopyranose mutase that catalyzes the formation of UDP-l-arabinofuranose from UDP-l-arabinopyranose. So, the content of Ara should be increased. Furthermore, the activities of UDP-glucose 4,6-dehydratase and 3,5-epimerase/4-reductase were decreased, thus reducing Rha. Owing to the downregulation in hexokinase and xylan 1,4-beta-xylosidase expression, Man and Xyl were reduced in the meantime. Furthermore, UDP-glucuronate 4-epimerase contributes to the dimerization of pectin rhamnogalacturonan-II,^45^ and the lower expression results in low pectin content in the cell wall. In the 76 *vs.* 55 DAF group, Ara was accumulated, whereas Rha, Man, and Xyl were decreased. Meanwhile, the expression of UDP-glucuronate 4-epimerase was decreased as well. These results propose that cell wall synthesis is slower and cell division was slowed down. Therefore, with the fruit at the 76 DAF stage, the jujube fruit puffing slowed down and attained a stable growth stage.

The redox process of ascorbate is closely related to the growth and development of plants and has regulatory effects on the cell cycle. Studies have also shown that ascorbate promotes the cell cycle, whereas oxidative ascorbic acid (DHA) hinders normal cell cycle progression.^46^ Additionally, ascorbate is involved in regulating cell division and elongation and participates in the synthesis and elongation of the cell wall.^47^ Formation of GDP-l-galactose from GDP-d-mannose is catalyzed by GDP-man-nose-3′,5′-epimerase (GME), which is the first step in plant ascorbate biosynthesis at the glucoside level. This step is also the key link in ascorbate feedback regulation. Different redox states of cells affect this regulation, including internal and external environmental stress conditions. GME is one of the enzymes that convert from GDP-d-mannose and GDP-l-gulose to l-galactose. l-Galactose, the last precursor in ascorbic acid synthesis, promotes ascorbic acid synthesis in tobacco cells while improving cell mitotic index.^48^ Ascorbate oxidase (AO) and ascorbate peroxidase (APX) are two main oxidases that catalyze the decomposition of ascorbate. Ascorbate can also be oxidized to form monodehydroascorbate (MDA), which produces ascorbate and dehydrogenated ascorbic acid (DHA) by disproportionation without enzyme catalysis. Alternatively, MDA reacts with NADH to produce ascorbate as catalyzed by monodehydroascorbate reductase (MDHAR). Studies have shown that increased levels of MDA outside the cytoplasm promoted cell elongation, which was related to high expression levels of AO.^48^ During the fruit-puffing period, fruit suffer stronger oxidation stress; however, during stable growth periods, oxidation stress is mitigated. In this study, GME, AO, and APX were downregulated, whereas MDHAR was upregulated, which indicated that ascorbate production decreases gradually when jujube fruit attains a stable growth stage.

As shown in Table 1 and Fig. 7B, C, *MDAR*1, *RHM*1, and *NRS*/*ER* are identified. In Table 1, *MDAR*1 is involved in ascorbate and aldarate metabolism, and *RHM*1 and *NRS*/*ER* are involved in amino and nucleotide sugar metabolism. As illustrated in Fig. 7B, *GPX*1, *FSD*2, and *APX*3 are connected to *MDAR*1 directly, whereas *BCCP*2, *At*1*g*24360, *EMB*3147, and *At*5*g*64250 are connected to *MDAR*1 indirectly. A possible fact is that *GPX*1, *FSD*2, and *APX*3 are closely related to ascorbate and aldarate metabolism and act on jujube. *GLC*, *GME*, and *TT*4 are also connected to *RHM*1 and *NRS*/*ER* directly. Similarly, *TT*2 and *TT*10 are connected to *TT*4 directly as well. All results propose that *GLC*, *GME*, *TT*4, *RHM*1, and *NRS*/*ER* are associated with amino and nucleotide sugar metabolism and that *TT*2 and *TT*10 are associated with other KEGG pathways in the 76 *vs.* 55 DAF group.

### 4.2 Proteins associated with energy metabolism and production of phenolic compound

Jujube flavor is a combination of volatile organic compounds, which are synthesized during ripening and characterize a specific fruit or variety.^49^ As is the case with many other fruits, volatiles are derived from nutritionally important metabolites such as essential amino acids, fatty acids, and carotenoids.^50^ In most plant tissues, the biosynthesis of fatty acids, in which acetyl-CoA is converted to C_16_ fatty acids, occurs in the cytosol. Also, a heterogeneous group of molecules with saturated and unsaturated, straight-chain, branched-chain, and cyclic structures bearing various functional groups such as alcohols, aldehydes, ketones, and esters, is found as well in fresh jujube fruits.^51^

In higher plants, the amino acid phenylalanine (Phe) is a substrate of phenylpropanoid metabolism, a network of secondary metabolic pathways in the cytosol.^52,53^ Plants use Phe as a precursor to synthesize various phenolic natural products, including diverse and abundant phenylpropanoids such as anthocyanins, flavonoids, isoflavonoids, tannins, and volatiles.^54,55^ Most phenylpropanoids are also related to fruit coloring. Phe is an aromatic amino acid required for protein synthesis in plants.^53^ Yet in many cases, more phenylalanine is allocated to the phenylpropanoid metabolism than to protein biosynthesis.^52^

Fatty acid biosynthesis (ESI Fig. 3†) and phenylalanine metabolism (ESI Fig. 4†) were enriched in the 96 *vs.* 76 DAF group. The most important process in fatty acid biosynthesis is the formation of malonyl-CoA under the action of acetyl-CoA carboxylase (ACCase). Here malonyl-CoA, in turn, acts as a building block for fatty acid elongation. ACCase is composed of a biotin carboxyl carrier protein (BCCP). Also, three subunits, namely, biotin carboxylase (BC), BCCP, and carboxyl transferase (CT), were linked with BCCP.^56^ In our study, these enzymes, BC, BCCP, and CT, were significantly increased, suggesting that malonyl-CoA synthesis was promoted.

In higher plants, the acyl-carrier-protein (ACP) is an important cofactor in fatty acid biosynthesis,^57^ which is closely related to the content, composition, and proportion of unsaturated fatty acid storage by plants. During the synthesis of long-chain fatty acids, the starting unit is accepted to form the ACP complex, which is then transferred to the long chain of macromolecules that need to be extended. The expression of [ACP] *S*-malonyltransferase is thus increased, which then catalyzes the synthesis of the malonyl-[ACP] by the ACP and malonyl-CoA. Subsequently, 3-oxoacyl-[ACP] synthase II and 3-oxoacyl-[ACP] reductase, which are associated with the synthesis of long-chain fatty acid, was significantly increased in this group. The fatty acyl-ACP thioesterase B is also involved in the elongation of fatty acid chains. Alternatively, long-chain acyl-CoA synthetase participates in the beta-oxidation process of fatty acids. Furthermore, numerous fatty acid synthase subunits, which catalyze the carbon chain extension, were upregulated in the 96 DAF group, showing that fatty acids were rapidly synthesized around 96 DAF. This result correlates with the high demand for lipid biosynthesis during fruit ripening, which participates in the formation of jujube aroma as a precursor and provides a storage matrix for the accumulating carotenoids.^14^ Additionally, compared with all hub genes, in the 96 *vs.* 76 DAF group (Fig. 7e and Table 1), identified common genes were *FaTA*, *At*1*g*24360, *EMB*3147, *CAC*3, and *CAC*2. Furthermore, *P*5*CS*1, *ACC*1, *FAB*1, and *E*1-*BETA*-1 were closely related to those common genes. This result indicates that this hub module is mainly involved in fatty acid biosynthesis and proposes that *P*5*CS*1, *ACC*1, *FAB*1, and *E*1-*BETA*-1 are also associated with fatty acid biosynthesis.

Phenylalanine ammonia lyase was downregulated, illustrating that the content of phenylalanine was increased. Therefore, more phenylalanine flowed into the phenylpropanoid biosynthesis, preparing jujube for color changes. According to a recent report, the content of phenylalanine increased gradually with the coloring of the pericarp,^58^ and aspartate aminotransferase (AAT) can interact with the amino acid Phe and phenylpyruvate.^59^ AAT were up-expressed in “96 *vs.* 76 DAF” group, which indicated that metabolism was active between phenylalanine and phenylpyruvate. Additionally, a previous study showed that phenylacetaldehyde (PHA) and phenylethylalcohol are aromatic aroma compounds directly catalyzed by phenylalanine lyase. These are important scent compounds in numerous fruits. l-Tryptophan decarboxylase catalyzes the synthesis of phenylethylamine, and then PHA is synthesized by primary amine oxidase. The expression of the primary amine oxidase was increased, in 96 DAF, which proposes that aromatic component-synthesis was accelerated. Furthermore, 3-hydroxybutyryl-CoA dehydrogenase was upregulated, resulting in the increased synthesis of acetyl-CoA and succinyl-CoA, and an increased entry into the tricarboxylic acid cycle. This result proposes that the growth of jujube fruit needed more energy, which was related to the biosynthesis of secondary metabolites as identified in 96 DAF.

### 4.3 Proteins associated with color changes

High-quality fruit typically have deep red coloration, high sugar content, and a balanced sugar-to-acid ratio.^60,61^ The color change of jujube fruit depends on the content and proportion of chlorophyll, carotenoids, flavonoid, and anthocyanins in the fruit peel.^62^ The type and content in the pigment also determines the color of jujube fruit. The green and yellow colors of the fruit epidermis are derived from chlorophyll and carotenoids as well, whereas the red and reddish-purple coloration is mainly determined by the content and proportion of phenolic acid, flavonoids, flavanols, and anthocyanins, which are associated with the antioxidant activity of jujube fruit.^63^

Photosynthesis (ESI Fig. 5†) and flavonoid biosynthesis (ESI Fig. 6†) pathways were associated with fruit color changes and were enriched in the 116 DAF *vs.* 96 DAF group. Converting chloroplast into chromoplast is an important part of the ripening process and is typically associated with dismantling the photosynthetic machinery and accumulating carotenoids in chromoplasts.^64^ Also, chlorophyll is a key photosynthetic pigment in plant chloroplasts.^65^ During the light reaction, the thylakoid membrane of chloroplasts absorbs light energy, which decomposes water and releases oxygen. This membrane then stores light energy in the form of ATP and NADPH through the photosynthetic electron transport chain. The photosynthetic electron transport chain is composed of four complexes on the thylakoid membrane: the photosynthetic system II(PSII), cytochrome b6/f complex (Cyt b6/f), photosynthetic system (PSI), and ATP synthase complex (ATPase). The Cyt b6/f, located between PSII and PSI in the photosynthetic electron transport chain, is a plastoquinone (PQH2)/plastoblue (PC) oxidoreductase. In contrast, Cyt b6f plays a key role in electron transfer and energy conversion during photosynthesis. First, it acts as an electron carrier to mediate electron transfer between PSII and PSI. Furthermore, it is used as an energy converter to transfer protons from the outer side of the thylakoid membrane to its inner side using the electron-free energy released in the process of electron transport. This step results in the formation of a transmembrane proton electrochemical gradient, which provides power for ATP synthesis by ATP synthase. From the results, 10 DEPs mainly related to the photosynthesis pathway, that is, four proteins related to photosystem I (PSI), four related to photosystem II (PSII), and two related to the cytochrome b6/f complex, were identified. It means that jujube decreased photosynthesis by decreasing their enzyme activity in the 116 *vs.* 96 DAF group. The decrease in photosynthesis intensity should be related to the decrease in chlorophyll content in the process of jujube peel color progression from semi-red to full red. In this stage, the energy required for jujube metabolism is proposed to be reduced by photosynthesis. Additionally, as shown in Table 1 and Fig. 7f, *PSBR* and *DRT*112 were common genes. Studies have shown that *PSBR* and *DRT*112 are involved in photosynthesis. According to the edge color, most nodes were closely related to *ACP*4, *PSBQ*-2, *PSAE*-2, *PETC*, *SBPASE*, *PSAH*2, *NPQ*4, *GAPB*, *PSAK*, *AOR*, *ENH*1, and *PSBP*-1. This result indicates that these hub genes led to the fruit changes observed during this period (116 *vs.* 96 DAF group).

Flavonoids are a series of natural compounds that are widely distributed in plants and play important roles in diverse BPs such as imparting various colors, as they are major pigmentation factors.^35^ Various flavonoid substances have been reported in jujube fruit, including catechins, epicatechins, naringenin, and other flavanols.^66^ In the 116 *vs.* 96 DAF group, it was observed that the content of flavanols or flavonols increased by promoting the process of phenylpropanoid metabolism in the early stage of ripening. The expression of these key enzymes such as chalcone synthase, cinnamate-4-hydroxylase (C4H), flavanone 4-reductase, anthocyanidin reductase, and chalcone isomerase, were, however, decreased, which indicated that the content of flavonoids in jujube fruit decreased from 96 DAF to 116 DAF. Previous studies by X. Li *et al.* also had the same conclusion.^66^

## 5 Conclusion

To better understand the change of melatonin in jujube fruit developmental and ripening, a LFQ proteomic tool was used to investigate the expression proteins present during the developmental and ripening stages (55, 76, 96, and 116 DAF). Results showed that physiological traits of the seedless jujube fruits varied significantly at four growth stages, with a remarkable correlation with fruit quality changes. From the study, 262, 315, and 532 DEPs were identified in the 76 *vs.* 55 DAF, 96 *vs.* 76 DAF, and 116 *vs.* 96 DAF groups, respectively. Hierarchical clustering of stage-responsive proteins also revealed the metabolic changes during fruit growth. Similarly, GO and KEGG annotation and enrichment analysis of DEPs showed that the 76 *vs.* 55 DAF group pathways was amino and nucleotide sugar metabolic pathways, including those related to ascorbate and aldarate metabolism. These pathways were associated with cell division and resistance. Fatty acid biosynthesis and phenylalanine metabolism was mainly enriched in the 96 *vs.* 76 DAF group. Also, aromatic compounds were synthesized in large quantities at this stage. During the ripening stage, 55 DEPs were involved in photosynthesis and flavonoid biosynthesis. In the 116 *vs.* 96 DAF group as well, the color of jujube turned red, flavonoids were produced in large quantities, and photosynthesis was significantly reduced. Hence, our results established a link between protein profiles and growth phenotypes, which will help improve our understanding of seedless jujube fruit growth at the proteomic level. Our results also added information on proteome changes during ripening.

## Conflicts of interest

The authors declare that there are no competing financial interests.

## Supplementary Material

RA-011-D1RA02989D-s001

## References

[cit1] Forestry Statistical Yearbook 2016, ed J. L. Zhang and C. L. Li, China Forestry Publishing House, Beijing, China, 2017, vol. 70, pp. 83–87

[cit2] Rahman E., Momin A., Zhao L., Guo X., Xu D., Zhou F., Ji B. (2018). Bioactive, nutritional composition, heavy metal and pesticide residue of four Chinese jujube cultivars. Food Sci. Biotechnol..

[cit3] Chen J. P., Li Z. J., Maiwulanjiang M., Zhang W. L., Zhan J. Y. X., Lam C. T., Zhu K. Y., Yao P., Choi R. C. Y., Lau D. T., Dong T. T. X., Tsim K. W. K. (2013). Chemical and biological assessment of *Ziziphus jujuba* fruits from China: different geographical sources and developmental stages. J. Agric. Food Chem..

[cit4] Du L. J., Gao Q. H., Ji X. L., Ma Y. J., Xu F. Y., Wang M. (2013). Comparison of flavonoids, phenolic acids, and antioxidant activity of explosion-puffed and sun-dried jujubes (*Ziziphus jujuba* Mill.). J. Agric. Food Chem..

[cit5] Zhang Q., Wang L. L., Liu Z. G., Zhao Z. H., Wang Z. T., Zhou G. F., Liu P., Liu M. J. (2020). Transcriptome and metabolome profiling unveil the mechanisms of *Ziziphus jujuba* Mill. peel coloration. Food Chem..

[cit6] Chen J. P., Liu X. Y., Li Z. G., Qi A. R., Yao P., Zhou Z. Y., Dong T. T. X., Tsim K. W. K. (2017). A Review of Dietary *Ziziphus jujuba* Fruit (Jujube): Developing Health Food Supplements for Brain Protection. J. Evidence-Based Complementary Altern. Med..

[cit7] Chen J. P., Tsim K. W. K. (2020). A Review of Edible Jujube, the *Ziziphus jujuba* Fruit: A Heath Food Supplement for Anemia Prevalence. Front. Pharmacol..

[cit8] Shi Q. Q., Zhang Z., Su J. J., Zhou J., Li X. G. (2018). Comparative Analysis of Pigments, Phenolics, and Antioxidant Activity of Chinese Jujube (*Ziziphus jujuba* Mill.) during Fruit Development. Molecules.

[cit9] D'Ambrosio C., Arena S., Rocco M., Verrillo F., Novi G., Viscosi V., Marra M., Scaloni A. (2013). Proteomic analysis of apricot fruit during ripening. J. Proteomics.

[cit10] Shi Q. Q., Du J. T., Zhu D. J., Li X., Li X. G. (2020). Metabolomic and Transcriptomic Analyses of Anthocyanin Biosynthesis Mechanisms in the Color Mutant *Ziziphus jujuba* cv. Tailihong. J. Agric. Food Chem..

[cit11] Liu X., Wang T. Y., Chen L., Li L. M., Wang Y., Li X. H., Xing Y. G. (2018). Transcriptomic and gene expression changes in response to postharvest surface pitting in ‘Lingwu Long’ jujube fruit. Hortic., Environ. Biotechnol..

[cit12] Chen X., Chen R. H., Wang Y. F., Wu C. Y., Huang J. (2019). Genome-Wide Identification of WRKY Transcription Factors in Chinese jujube (*Ziziphus jujuba* Mill.) and Their Involvement in Fruit Developing, Ripening, and Abiotic Stress. Genes.

[cit13] Wu X. Q., Jiang L., Yu M. L., An X. J., Ma R. J., Yu Z. F. (2016). Proteomic analysis of changes in mitochondrial protein expression during peach fruit ripening and senescence. J. Proteomics.

[cit14] Sun Q. Q., Zhang N., Wang J. F., Cao Y. Y., Li X. S., Zhang H. J., Zhang L., Tan D. X., Guo Y. D. (2016). A label-free differential proteomics analysis reveals the effect of melatonin on promoting fruit ripening and anthocyanin accumulation upon postharvest in tomato. J. Pineal Res..

[cit15] Zheng Q. F., Song J., Campbell-Palmer L., Thompson K., Li L., Walker B., Cui Y. S., Li X. H. (2013). A proteomic investigation of apple fruit during ripening and in response to ethylene treatment. J. Proteomics.

[cit16] Shi Y., Jiang L., Zhang L., Kang R., Yu Z. F. (2014). Dynamic changes in proteins during apple (*Malus x domestica*) fruit ripening and storage. Hortic. Res..

[cit17] Li X., Bi Y., Wang J. J., Dong B. Y., Li H. J., Gong D., Zhao Y., Tang Y. M., Yu X. Y., Shang Q. (2015). BTH treatment caused physiological, biochemical and proteomic changes of muskmelon (*Cucumis melo* L.) fruit during ripening. J. Proteomics.

[cit18] Du W., Xiong C. W., Ding J., Hilde N., Ruan C. J., Guo H. (2019). Tandem Mass Tag Based Quantitative Proteomics of Developing Sea Buckthorn Berries Reveals Candidate Proteins Related to Lipid Metabolism. J. Proteome Res..

[cit19] Zhang W. T., Li X. H., Li L., Tang Y., Qi W., Liu X., Qiao L. P., Wang W., Jia X. Y. (2017). A label-free quantitative proteomic investigation reveals stage-responsive ripening genes in apricot. J. Hortic. Sci. Biotechnol..

[cit20] Zheng J. S., Wei R. Y., Wang Z., Zhu T. T., Ruan H. R., Wei X., Hou K. W., Wu R. (2020). Serum proteomics analysis of feline mammary carcinoma based on label-free and PRM techniques. J. Vet. Sci..

[cit21] Fabre B., Korona D., Groen A., Vowinckel J., Gatto L., Deery M. J., Ralser M., Russell S., Lilley K. S. (2016). Analysis of drosophila melanogaster proteome dynamics during embryonic development by a combination of label-free proteomics approaches. Proteomics.

[cit22] Megger D. A., Pott L. L., Ahrens M., Padden J., Bracht T., Kuhlmann K., Eisenacher M., Meyer H. E., Sitek B. (2014). Comparison of label-free and label-based strategies for proteome analysis of hepatoma cell lines. Biochim. Biophys. Acta.

[cit23] Neilson K. A., Ali N. A., Muralidharan S., Mirzaei M., Mariani M., Assadourian G., Lee A., van Sluyter S. C., Haynes P. A. (2011). Less label, more free: approaches in label-free quantitative mass spectrometry. Proteomics.

[cit24] Venable J. D., Dong M. Q., Wohlschlegel J., Dillin A., Yates J. R. (2004). Automated approach for quantitative analysis of complex peptide mixtures from tandem mass spectra. Nat. Methods.

[cit25] Carvalho P. C., Han X. M., Xu T., Cociorva D., da Gloria Carvalho M., Barbosa V. C., Yates III J. R. (2010). XDIA: improving on the label-free data-independent analysis. Bioinformatics.

[cit26] Li L., Luo Z. S., Huang X. H., Zhang L., Zhao P. Y., Ma H. Y., Li X. H., Ban Z. J., Liu X. (2015). Label-free quantitative proteomics to investigate strawberry fruit proteome changes under controlled atmosphere and low temperature storage. J. Proteomics.

[cit27] Buts K., Michielssens S., Hertog M. L., Hayakawa E., Cordewener J., America A. H., Nicolai B. M., Carpentier S. C. (2014). Improving the identification rate of data independent label-free quantitative proteomics experiments on non-model crops: a case study on apple fruit. J. Proteomics.

[cit28] Katz E., Fon M., Eigenheer R. A., Phinney B. S., Fass J. N., Lin D. W., Sadka A., Blumwald E. (2010). A label-free differential quantitative mass spectrometry method for the characterization and identification of protein changes during citrus fruit development. Proteome Sci..

[cit29] Pocsfalvi G., Turiák L., Ambrosone A., del Gaudio P., Puska G., Fiume I., Silvestre T., Vékey K. (2018). Protein biocargo of citrus fruit-derived vesicles reveals heterogeneous transport and extracellular vesicle populations. J. Plant Physiol..

[cit30] Hou L., Zhang Z. Y., Dou S. H., Zhang Y. D., Pang X. M., Li Y. Y. (2019). Genome-wide identification, characterization, and expression analysis of the expansin gene family in Chinese jujube (*Ziziphus jujuba* Mill.). Planta.

[cit31] Zheng J. S., Wei R. Y., Wang Z., Zhu T. T., Ruan H. R., Wei X., Hou K. W., Wu R. (2020). Serum proteomics analysis of feline mammary carcinoma based on label-free and PRM techniques. J. Vet. Sci..

[cit32] Jia W., Shi Q. Y., Zhong R., Shi L., Chu X. G. (2021). Unraveling proteome changes of irradiated goat meat and its relationship to off-flavor analyzed by high-throughput proteomics analysis. Food Chem..

[cit33] Soares E. A., Werth E. G., Madroñero L. J., Ventura J. A., Rodrigues S. P., Hicks L. M., Fernandes P. M. (2017). Label-free quantitative proteomic analysis of pre-flowering PMeV-infected Carica papaya L. J. Proteomics.

[cit34] Wang S. S., Cai Y., Cheng J. Q., Li W. X., Liu Y. S., Yang H. (2019). motifeR: An Integrated Web Software for Identification and Visualization of Protein Post-Translational Modification Motifs. Proteomics.

[cit35] Wang C., Wang J., Wang X., Xia Y., Chen C., Shen Z. G., Chen Y. H. (2017). Proteomic analysis on roots of oenothera glazioviana under copper-stress conditions. Sci. Rep..

[cit36] Sun N., Sun W., Li S., Yang J., Yang L., Quan G., Gao X., Wang Z., Cheng X., Li Z., Peng Q., Liu N. (2015). Proteomics Analysis of Cellular Proteins Co-Immunoprecipitated with Nucleoprotein of Influenza A Virus (H7N9). Int. J. Mol. Sci..

[cit37] Ye J. Z., Su Y. B., Lin X. M., Lai S. S., Li W. X., Ali F., Zheng J., Peng B. (2018). Alanine Enhances Aminoglycosides-Induced ROS Production as Revealed by Proteomic Analysis. Front. Microbiol..

[cit38] Sun G., Zhang S., Zhang Y., Xu K., Zhang Q., Zhao T., Zheng X. (2019). Effective Dimensionality Reduction for Visualizing Neural Dynamics by Laplacian Eigenmaps. Neural Comput..

[cit39] Bhargab K., Shehnaz B., Maleppillil V. V., Khushman T., Vasudevan S., Srikanth R. (2020). Application of mass spectrometry based proteomics to understand diabetes: a special focus on interactomics. Biochim. Biophys. Acta, Proteins Proteomics.

[cit40] Vieira Parrine Sant'Ana D., Lefsrud M. (2018). Tomato proteomics: tomato as a model for crop proteomics. Sci. Hortic..

[cit41] Liu Z. B., Lv J. H., Zhang Z. Q., Li H., Yang B. Z., Chen W. C., Dai X. Z., Li X. F., Yang S., Liu L., Ou L. J., Ma Y. Q., Zou X. X. (2019). Integrative Transcriptome and Proteome Analysis Identifies Major Metabolic Pathways Involved in Pepper Fruit Development. J. Proteome Res..

[cit42] Graham N., Foyer Christine H. (1998). Ascorbate and glutathione: keeping active oxygen under control. Annu. Rev. Plant Physiol. Plant Mol. Biol..

[cit43] Arrigoni O., Gara L. D., Paciolla C., Evidente A., de Pinto M. C., Liso R. (1997). Lycorine: a powerful inhibitor of L-galactono-gamma-lactone dehydrogenase activity. J. Plant Physiol..

[cit44] Wojdylo A., Carbonell-Barrachina Á. A., Legua P., Hernández F. (2016). Phenolic composition, ascorbic acid content, and antioxidant capacity of Spanish jujube (*Ziziphus jujube* Mill.) fruits. Food Chem..

[cit45] Mounet-Gilbert L., Dumont M., Ferrand C., Bournonville C., Monier A., Jorly J., Lemaire-Chamley M., Mori K., Atienza I., Hernould M., Stevens R., Lehner A., Mollet J. C., Rothan C., Lerouge P., Baldet P. (2016). Two tomato GDP-D-mannose epimerase isoforms involved in ascorbate biosynthesis play specific roles in cell wall biosynthesis and development. J. Exp. Bot..

[cit46] Potters G., De Gara L., Asard H., Horemans N. (2002). Ascorbate and glutathione: guardians of the cell cycle, partners in crime?. Plant Physiol. Biochem..

[cit47] Smirnoff N., Wheeler G. L., Loewus F. A. (2000). Ascorbic Acid in Plants: Biosynthesis and Function. Crit. Rev. Biochem. Mol. Biol..

[cit48] de Pinto M. C., Francis D., De Gara L. (1999). The redox state of the ascorbate-dehydroascorbate pair as a specific sensor of cell division in tobacco BY-2 cells. Protoplasma.

[cit49] Song J., Bi J., Chen Q., Wu X., Lyu Y., Meng X. (2019). Assessment of sugar content, fatty acids, free amino acids, and volatile profiles in jujube fruits at different ripening stages. Food Chem..

[cit50] Gonda I., Davidovich-Rikanati R., Bar E., Lev S., Jhirad P., Meshulam Y., Wissotsky G., Portnoy V., Burger J., Schaffer A. A., Tadmor Y., Giovannoni J. J., Fei Z., Fait A., Katzir N., Lewinsohn E. (2018). Differential metabolism of L-phenylalanine in the formation of aromatic volatiles in melon (*Cucumis melo* L.) fruit. Phytochemistry.

[cit51] Kuo T. M., Gardner H. W. (2002). The Palladion and Its Multiple Functions in the Parthenon North Metopes. Lipid Biotechnol..

[cit52] dams Z. P. A., hlting J. E., Edwards R. (2019). The regulatory role of shikimate in plant phenylalanine metabolism. J. Theor. Biol..

[cit53] Kaminaga Y., Schnepp J., Peel G., Kish C. M., Ben-Nissan G., Weiss D., Orlova I., Lavie O., Rhodes D., Wood K., Porterfield D. M., Cooper A. J., Schloss J. V., Pichersky E., Vainstein A., Dudareva N. (2006). Plant phenylacetaldehyde synthase is a bifunctional homotetrameric enzyme that catalyzes phenylalanine decarboxylation and oxidation. J. Biol. Chem..

[cit54] Maeda H. A. (2019). Harnessing evolutionary diversification of primary metabolism for plant synthetic biology. J. Biol. Chem..

[cit55] Maeda H., Dudareva N. (2012). The shikimate pathway and aromatic amino acid biosynthesis in plants. Annu. Rev. Plant Biol..

[cit56] Gerhardt E. C., Rodrigues T. E., Müller-Santos M., Pedrosa F. O., Souza E. M., Forchhammer K., Huergo L. F. (2015). The bacterial signal transduction protein GlnB regulates the committed step in fatty acid biosynthesis by acting as a dissociable regulatory subunit of acetyl-CoA carboxylase. Mol. Microbiol..

[cit57] Chen S. L. , Identification and functional analysis of lipid biosynthesis related genes in peanut (Arachis hypogaea L.), Chinese Academy of Agricultural Sciences Dissertation, 2012 (in Chinese)

[cit58] Zhang Q. , Analysis of peel structure and components related to pigment accumulation during jujube coloring, Hebei Agricultural University, Hebei, 2020

[cit59] Cárdenas-Fernández M., López C., Álvaro G., López-Santín J. (2012). L-Phenylalanine synthesis catalyzed by immobilized aspartate aminotransferase. Biochem. Eng. J..

[cit60] Chen M., Jiang Q., Yin X. R., Lin Q., Chen J. Y., Allan A. C., Xu C. J., Chen K. S. (2012). Effect of hot air treatment on organic acid and sugar-metabolism in Ponkan (Citrus reticulata) fruit. Sci. Hortic..

[cit61] Samykanno K., Pang E., Marriott P. J. (2013). Genotypic and environmental effects on flavor attributes of ‘Albion’ and ‘Juliette’ strawberry fruits. Sci. Hortic..

[cit62] Sarma B., Das K., Bora S. S. (2020). Physiology of Fruit Development. Int. J. Curr. Microbiol. Appl. Sci..

[cit63] Jiang W. Q., Li N., Zhang D. P., Lyndel M., Cao B., Li Y. J., Song L. H. (2020). Elevated temperature and drought stress significantly affect fruit quality and activity of anthocyanin-related enzymes in jujube (Ziziphus jujuba Mill. cv. ‘Lingwuchangzao’). PLoS One.

[cit64] Cocaliadis M. F., Fernández-Muñoz R., Pons C., Orzaez D., Granell A. (2014). Increasing tomato fruit quality by enhancing fruit chloroplast function. A double-edged sword?. J. Exp. Bot..

[cit65] Xiong B., Qiu X., Huang S. J., Wang X. J., Zhang X., Dong T. T., Li S. C., Sun G. C., Zhu J., Wang Z. H. (2019). Physiological and transcriptome analyses of photosynthesis and chlorophyll metabolism in variegated Citrus (Shiranuhi and Huangguogan) seedlings. Sci. Rep..

[cit66] Li X., Shi Q. Q., Zhu D. Y. (2020). *et al.*, The patterns of flavonoids accumulation and the expression of biosynthesis related genes during the course of maturation of the Chinese jujube fruit. J. Fruit Sci..

