# Supplementary material for: Label-free quantitative proteomics analysis of jujube (Ziziphus jujuba Mill.) during different growth stages
Source: RSC Adv. 2021 Jun 22;11(36):22106–19. doi: 10.1039/d1ra02989d (PMC9034241; doi:10.1039/d1ra02989d)
Supplement: RA-011-D1RA02989D-s001 [file RA-011-D1RA02989D-s001.pdf]

Electronic Supplementary Material (ESI) for RSC Advances.
This journal is © The Royal Society of Chemistry 2021

Electronic Supplementary Material (ESI) for RSC Advances.

Supplementary Fig.1 KEGG pathway mapping of amino sugar and nucleotide sugar metabolism

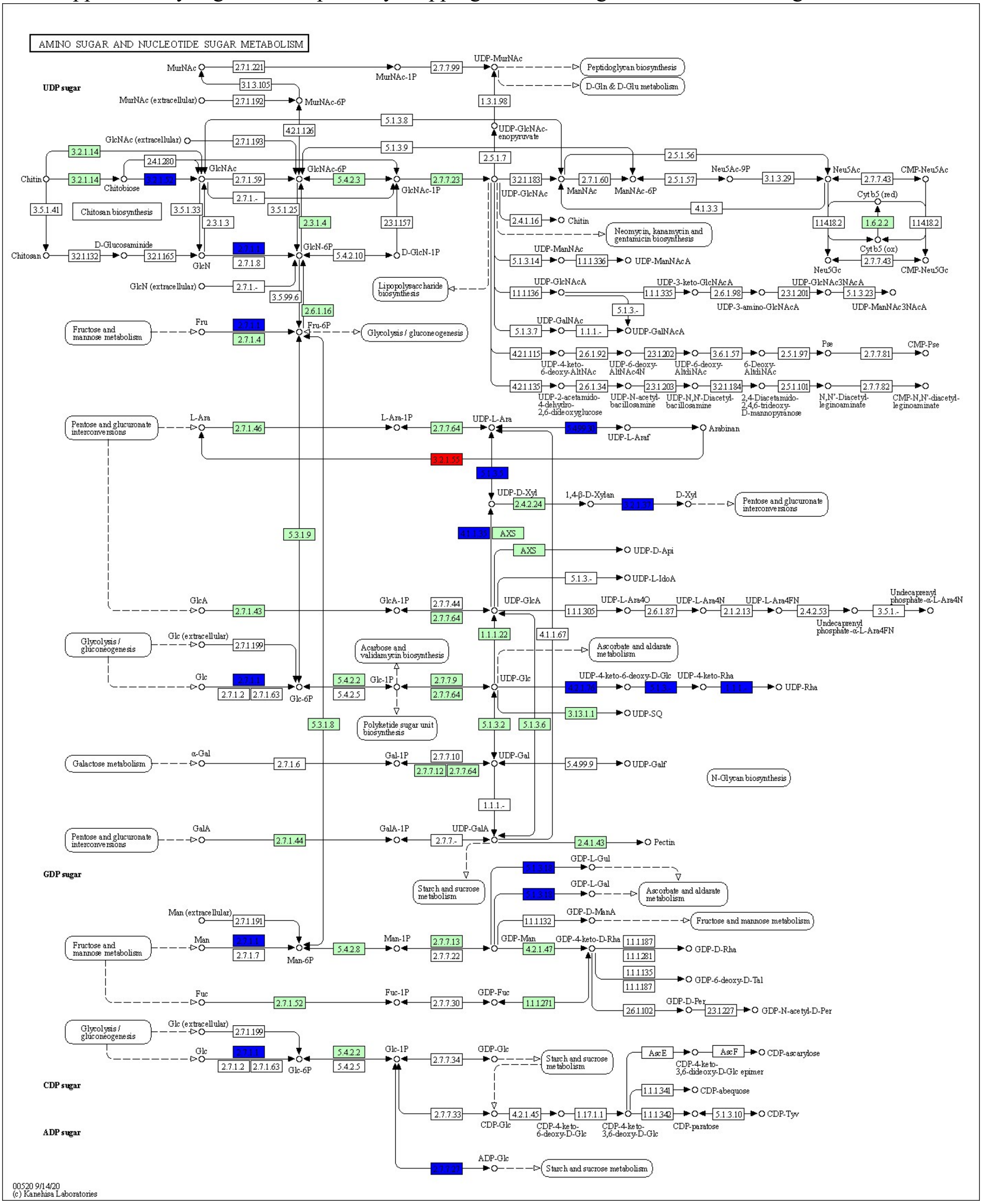

Box means gene or enzyme, and circle means chemical compound. Red represents up-regulation, while blue represents down-regulation. Green means particular gene or enzyme in this species.

Supplementary Fig.2 KEGG pathway mapping of ascorbate and aldarate metabolism

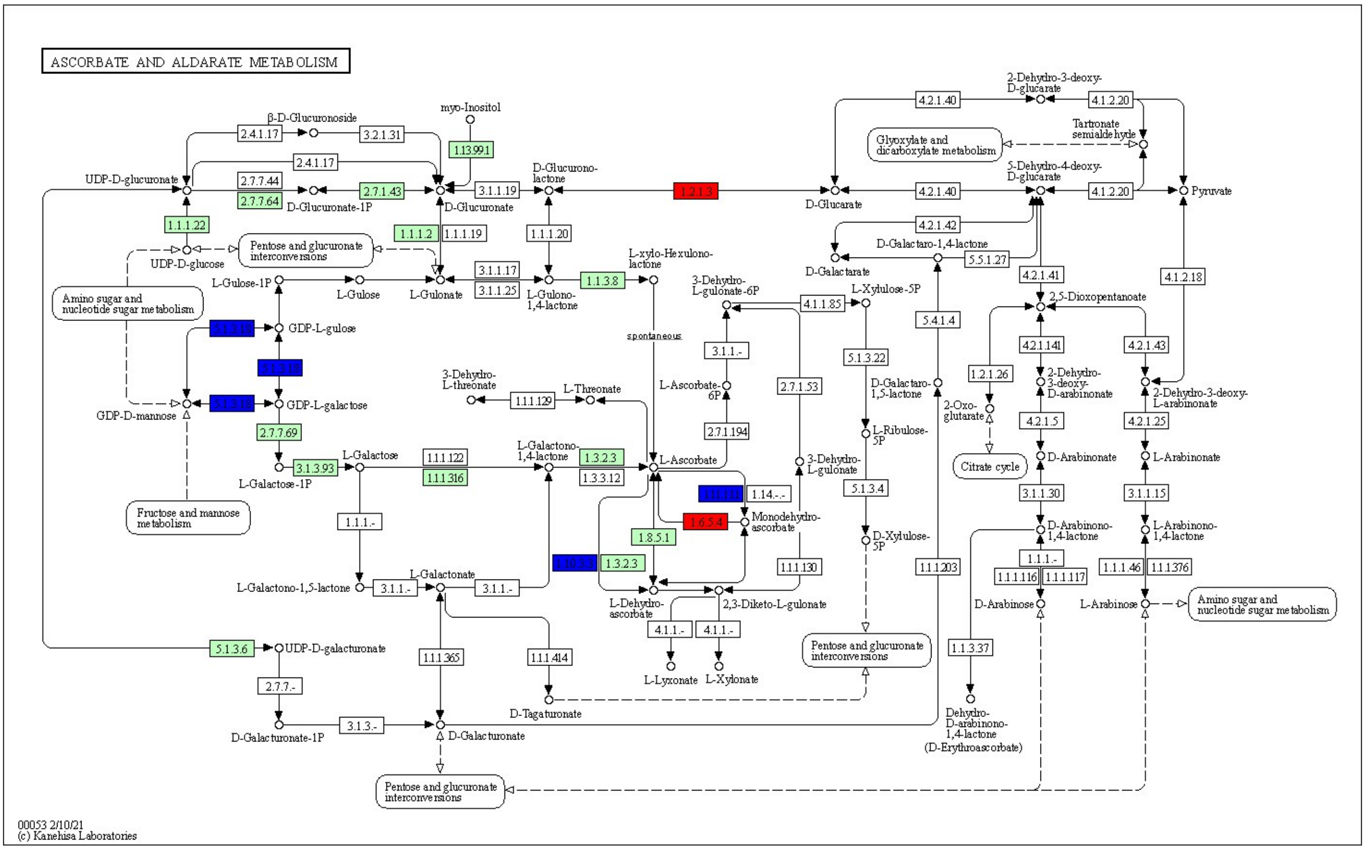

Supplementary Fig.3 KEGG pathway mapping of fatty acid biosynthesis

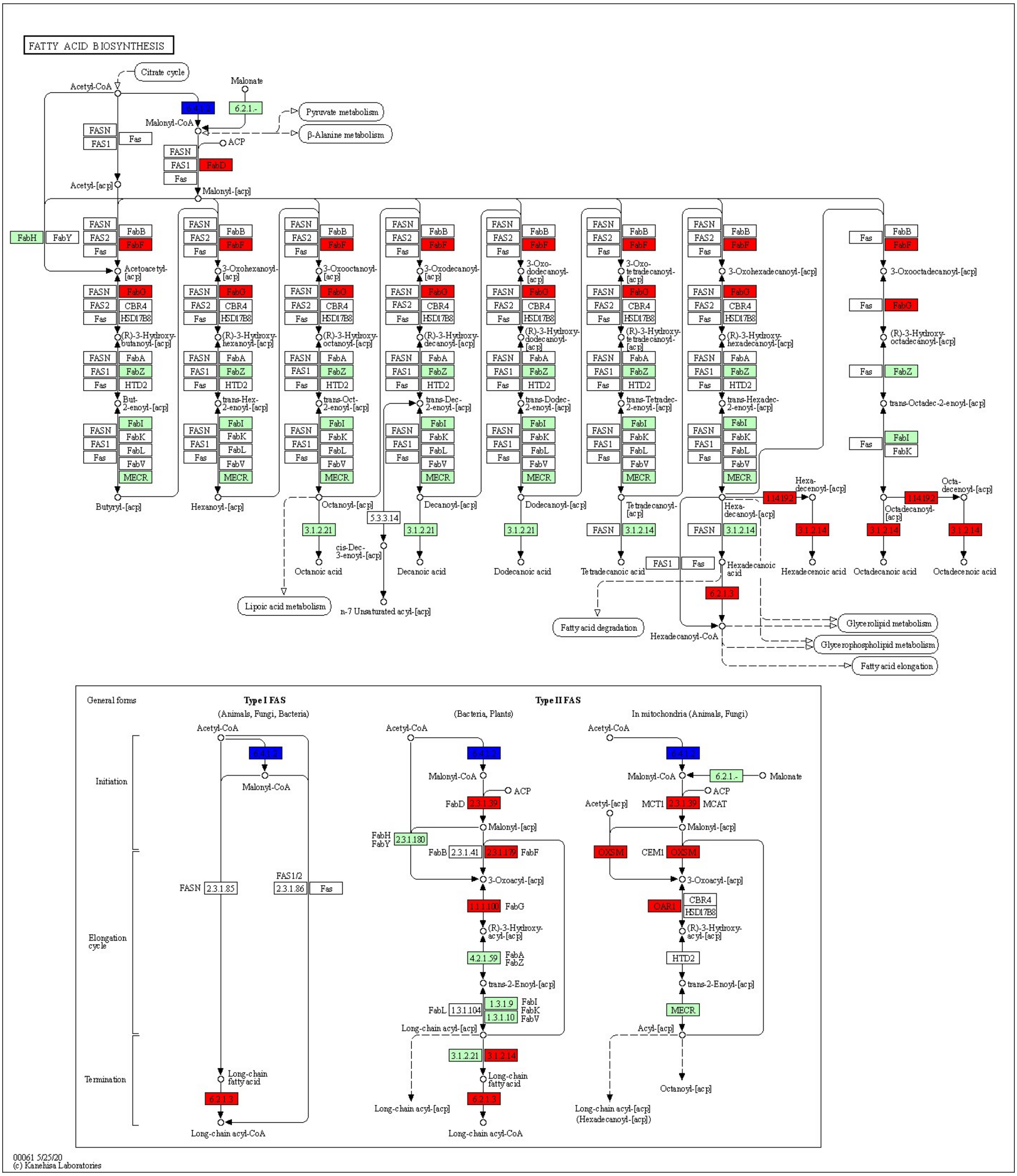

Supplementary Fig.4 KEGG pathway mapping of phenylalanine metabolism

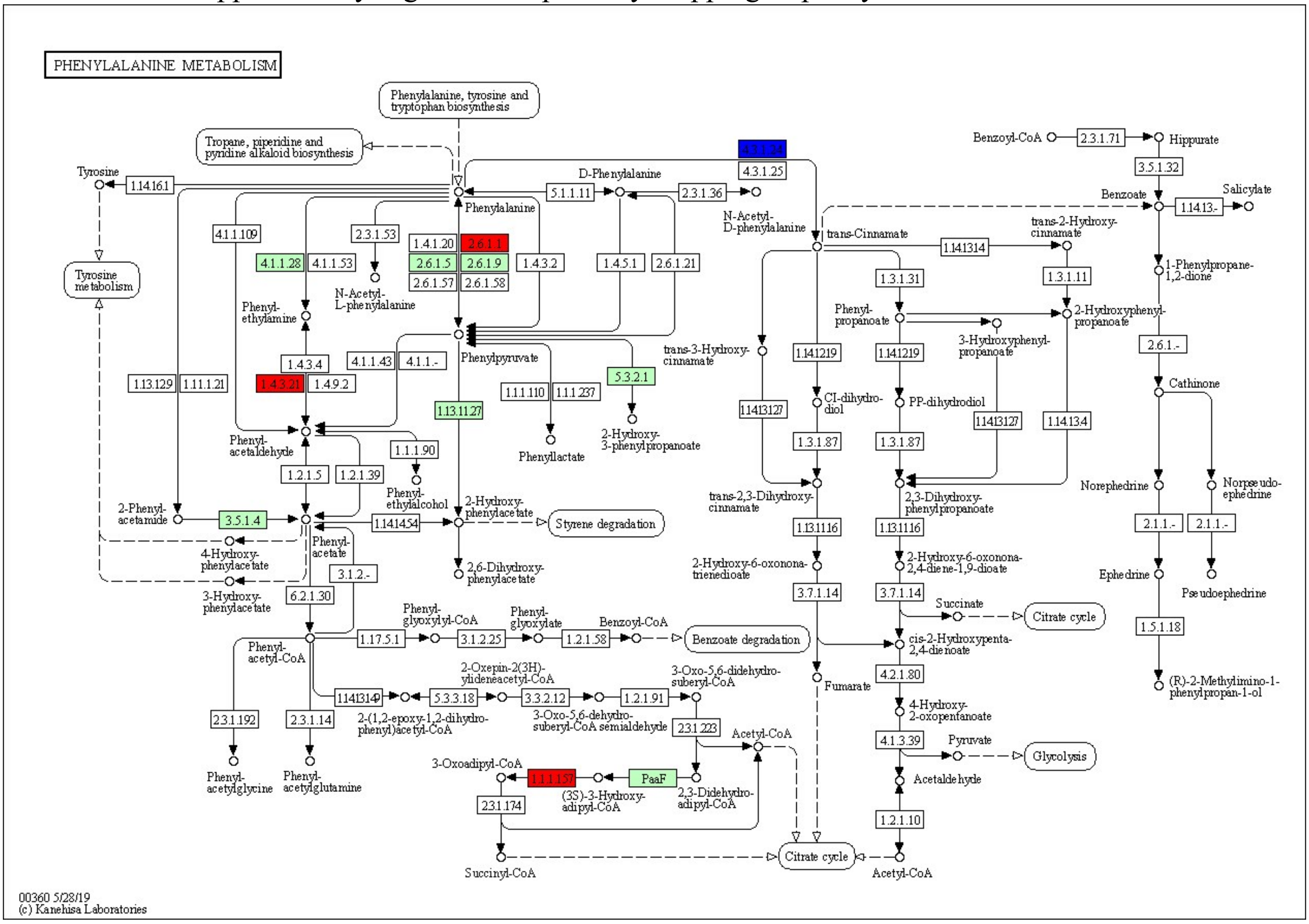

Supplementary Fig.5 KEGG pathway mapping of photosynthesis

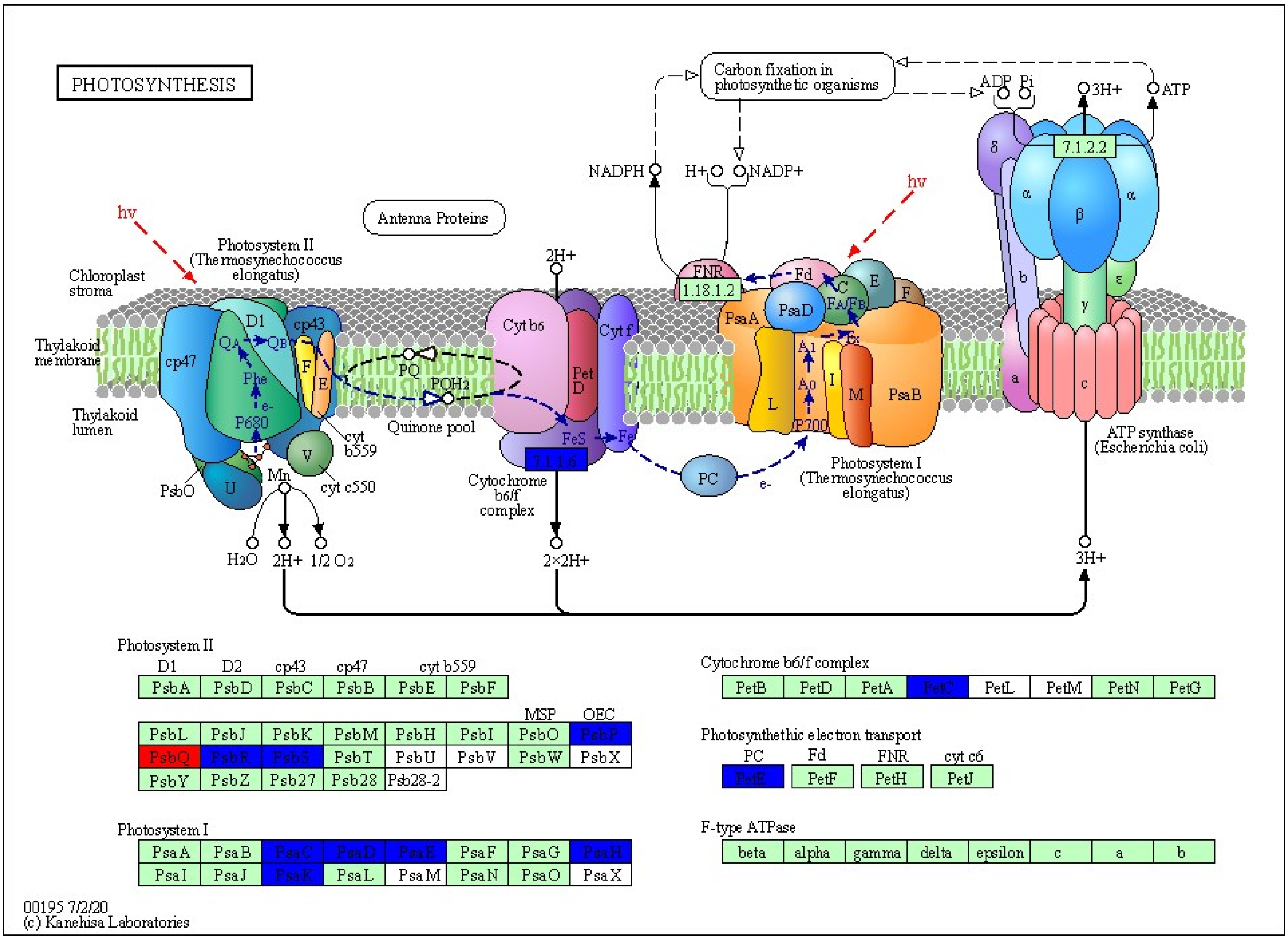

Supplementary Fig.6 KEGG pathway mapping of flavonoid biosynthesis.

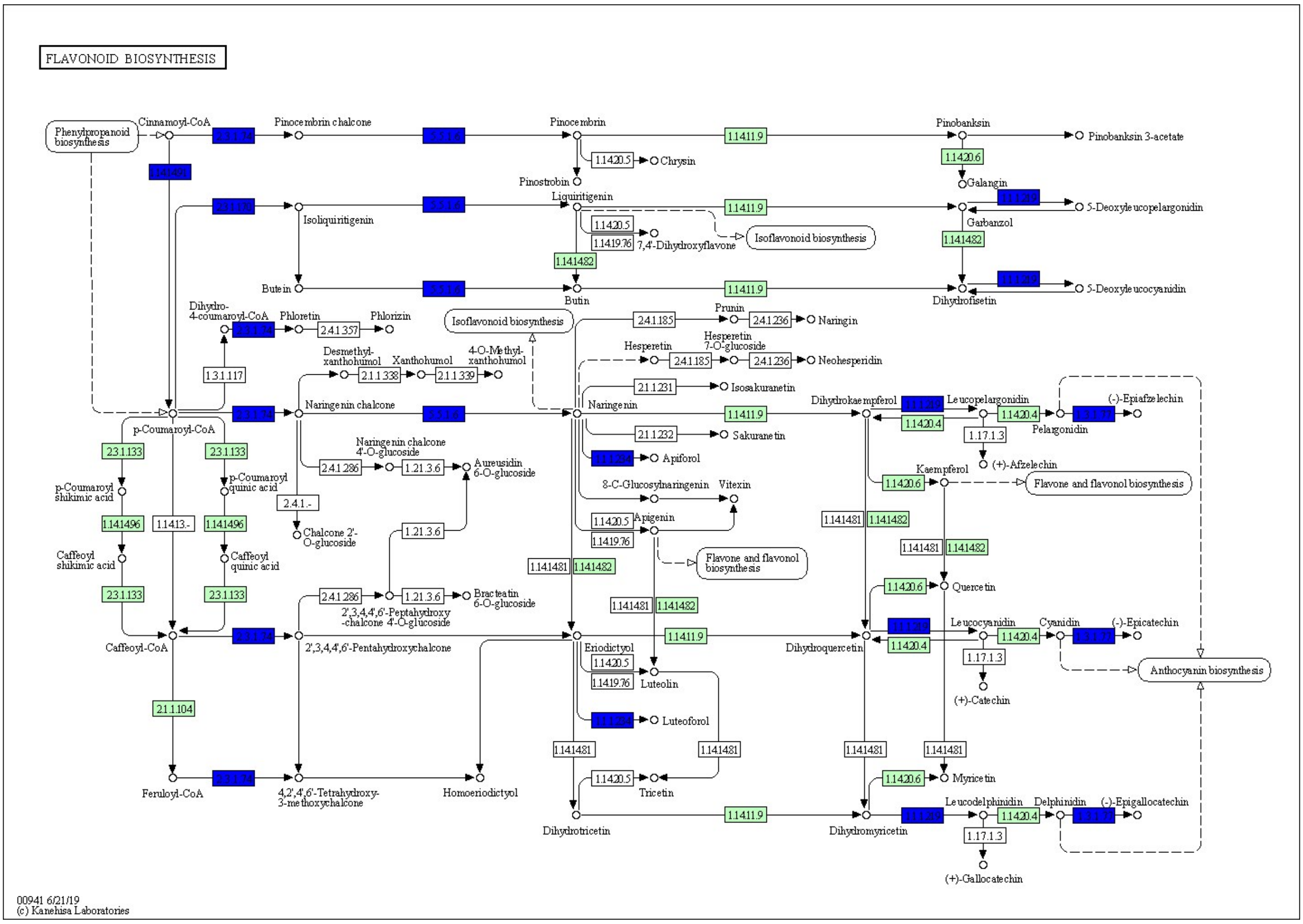

Supplementary Table 1 The top three enriched GO terms for DEPs in jujubes

Gene Ontology term		*P*-value adjusted	count
76 DAF vs 55 DAF			
	single-organism metabolic process	5.45E-22	135
Biological process	catabolic process	7.02E-12	62
	response to chemical	1.8E-09	69
	cell periphery	8.37E-20	74
Cellular component	plasma membrane	1.56E-10	49
	apoplast	7.91E-10	20
	oxidoreductase activity	1.63E-06	48
Molecular function	hydrolase activity	3.7E-06	77
	lyase activity	0.00641	14
96 DAF vs 76 DAF			
	single-organism metabolic process	1.35E-26	164
Biological process	response to abiotic stimulus	3.25E-15	67
	response to chemical	2.01E-14	90
	cell periphery	1.77E-19	85
Cellular component	intracellular part	1.12E-18	212
	intracellular	2.62E-16	214
	oxidoreductase activity	7.23E-09	60
Molecular function	carbohydrate transporter activity	3.91E-04	7
	protein binding	4.18E-04	57
116 DAF vs 96 DAF			
	response to abiotic stimulus	1.9E-30	118
Biological process	response to chemical	1.92E-25	149
	single-organism metabolic process	1.92E-25	228
	intracellular part	6E-40	357
Cellular component	intracellular	1.87E-39	366
	intracellular organelle part	1.09E-32	188
	protein binding	1.8E-16	120
Molecular function	oxidoreductase activity	8.16E-06	74
	peroxidase activity	1.42E-06	14
